# Anion-Exchange Chromatography at the Service of Gene Therapy: Baseline Separation of Full/Empty Adeno-Associated Virus Capsids by Screening of Conditions and Step Gradient Elution Mode

**DOI:** 10.3390/ijms232012332

**Published:** 2022-10-15

**Authors:** Megane K. Aebischer, Hugo Gizardin-Fredon, Honorine Lardeux, Dominik Kochardt, Carsten Elger, Markus Haindl, Raphael Ruppert, Davy Guillarme, Valentina D’Atri

**Affiliations:** 1 School of Pharmaceutical Sciences, University of Geneva, CMU—Rue Michel Servet 1, 1211 Geneva, Switzerland; 2 Institute of Pharmaceutical Sciences of Western Switzerland, University of Geneva, CMU—Rue Michel Servet 1, 1211 Geneva, Switzerland; 3 Roche Diagnostics GmbH, Nonnenwald 2, 82377 Penzberg, Germany

**Keywords:** anion-exchange chromatography, step gradient, gene therapy, recombinant adeno-associated virus, full/empty ratio

## Abstract

Gene therapy is opening unprecedented opportunities for novel therapeutic approaches. Based on the concept of rescuing function mutations by co-expressing the correct gene to allow biological functions to be restored, it requires the use of viral vectors to ensure the proper delivery of therapeutic genes. In this context, recombinant adeno-associated viruses (rAAV) are the most widely used vectors. Their biomanufacturing process requires the insertion of the therapeutic gene into the rAAV (full capsids). However, a percentage of rAAV that do not contain the desired gene (empty capsids), as well as partly filled capsids, might also be produced, potentially impacting the efficiency of the therapy. Therefore, the determination of the rAAV capsids’ full/empty ratio needs to be monitored to ensure consistent product quality and efficacy. Anion-exchange chromatography (AEX) can serve this need. In this contribution, thorough AEX method development, including a mobile phase, a stationary phase and gradient conditions, has highlighted its potential in supporting gene therapy. Taking advantage of the fact that viral capsids follow an “on/off” retention behavior, the application of a step gradient approach to the rAAV serotype 8 (rAAV8) allowed the unprecedented separation of rAAV8 full/empty capsids, with a resolution gain of 3.7 as compared to the resolution obtained with a fully optimized linear gradient. Finally, the developed analytical approach allowed a precise and accurate baseline separation and quantification of full and empty rAAV8 capsids, with the potential to be applied as a high-throughput quality control (QC) method.

## 1. Introduction

Gene therapy represents one of the greatest scientific advances in recent years and is opening extraordinary opportunities to treat or prevent genetic disorders linked to severe and rare diseases. This new therapeutic strategy is based on the concept that gene expression can be effectively modulated at the transcriptional level by replacing, inactivating or introducing genes into cells [1,2]. Supported by the scientific breakthroughs in the understanding of genotype-to-phenotype relationships, gene therapy allows the design of new therapeutic approaches based on “therapeutic gene delivery”. Indeed, to be effective, the therapeutic gene of interest (new or working copy of a missing or nonworking gene) needs to be properly delivered to human cells [3]. AAVs (adeno-associated viruses) have emerged as safe and attractive vectors for gene delivery [4], and recent approvals of AAV-based therapies by the Food and Drug Administration Agency (i.e., Luxturna in 2017 and Zolgensma in 2019) have confirmed that these delivery systems have a promising and bright future in the gene therapy era [5,6,7,8,9].

Twelve different AAV serotypes are known and characterized by specific target tissue tropism. An AAV is composed of the protein capsid which contains a viral genome. The capsid comprises around 60 copies of three viral proteins VP1, VP2, VP3 (at a 1:1:10 ratio, respectively) assembled into an icosahedron of 26 nm in diameter. The genome is composed of single-stranded DNA of about 4.8 kb. The structure of the genome is relatively simple as it is composed of three genes flanked by two inverted terminal repeats (ITRs) at the ends [10]. The first gene is the Rep gene which encodes four proteins (Rep78, Rep68, Rep52 and Rep40) produced from the same sequence but from different promoters and through alternative splicing [11]. They are useful for targeting viral integration, viral replication, transcription and packaging of viral DNA into the viral capsid [11]. The second gene is the Cap gene which encodes the three viral capsid proteins (VP1, VP2 and VP3) that are different despite the same genetic sequence, due to translation from different start codons and alternative splicing. The third gene encodes the assembly activation protein (AAP) and is located within the Cap coding sequence [11,12].

Given its simplicity, the AAV genome might be easily engineered to produce a recombinant AAV (rAAV) missing the viral DNA encoding the viral proteins (Rep and Cap). Indeed, the ITRs sequences of the viral genome are conserved to maintain the transcriptional activity, while the rest of the viral sequence is replaced by an expression cassette containing the therapeutic gene called a transgene [8]. The transgene allows the expression of a desired protein that might be, for example, a missing or nonfunctional protein related to a specific pathological state [13].

During the production of an rAAV, there is a risk of retaining impurities in the sample [14]. For example, the DNA may not be integrated properly in the capsids and give raise to the presence of empty capsids, which may correspond to ratios of 10% to 90% of total capsids [15]. Empty rAAVs and partially filled capsids are therefore product-related impurities that may reduce the effective concentration of the final drug, compete for binding sites and decrease the efficacy of the drug [16]. According to the FDA, these impurities need to be monitored and reported as the full/empty (F/E) ratio [17]. Because of the similarity to the desired AAV vector product, empty capsids are difficult to avoid or eliminate [18]. From an analytical point of view, they represent an issue as they have the same size as the full capsids and minor pI differences. Various analytical strategies were suggested to characterize empty capsid content, including electron microscopy, quantitative polymerase chain reaction (PCR), Elisa assay, UV absorbance spectrophotometry, analytical ultracentrifugation (AUC) or charge detection mass spectrometry (CDMS) [19,20]. In addition, as full rAAVs are loaded with negatively charged DNA within their structures, they have a slightly lower pI than empty rAAV capsids do (generally a difference in the range of 0.4 pH units), and this feature might be exploited in the attempt to separate and quantify full and empty capsids by anion-exchange chromatography (AEX) [21,22,23,24,25,26]. In this contribution, thorough AEX method development, including a mobile phase, a stationary phase, and gradient scouting, has highlighted the potential of the step gradient elution mode in supporting gene therapy. Taking advantage of the fact that viral capsids follow an “on/off” retention behavior [27,28], the application of a step gradient approach allowed the unprecedented separation of full/empty capsids related to rAAV serotype 8 (rAAV8). Finally, the developed analytical approach allowed a precise and accurate baseline separation and quantification of full and empty rAAV8 capsids, with the potential to be applied as a high-throughput quality control (QC) method.

## 2. Results and Discussion

### 2.1. Preliminary Selection of Column Hardware and Evaluation of Sample Stability

Generic salt-mediated AEX gradient conditions reported in Section 3.3.1 were used for a preliminary screening of different column hardware (Table 1). Four strong anion-exchanger adsorbents, all functionalized with quaternary ammonium and differing in terms of matrix composition (monolith or nonporous beads) and column material (stainless steel, PEEK or PEEK-lined stainless steel) were tested to determine their efficacy in resolving empty and full rAAV8 capsids. PP and PS are Thermo columns packed with either nonporous particles or monolith using PEEK housing, respectively. AX is an Agilent column packed with nonporous particles and PEEK housing, while the QS is a Tosoh column packed with nonporous particles. The latter is composed of stainless-steel tubing and PEEK frits. To work within the column pressure limits, the flow rate was decreased from 0.7 mL/min (used with PP and AX columns) to 0.5 and 0.35 mL/min when using the QS and PS columns, respectively. As reported in Figure S1, appropriate peak resolution (Rs) was obtained with the PP (Rs = 1.07) and PS (Rs = 1.00) columns. Based on these results, the PP column was selected for the rest of study. First, rAAV8 capsids’ stability was evaluated over time by considering freshly diluted samples prior to analysis and samples analyzed after 1, 3 or 7 days after dilution. In addition, the samples were diluted in either water or a PBS buffer enriched with 0.001% poloxamer 188 (generic formulation buffer) to also evaluate the impact of the dilution solvent on virus stability. As reported in Figure S2, samples diluted in water were quite unstable, showing remarkable variations of the peak areas already after 1 day, with a relative standard deviation (RSD) calculated over the 7 days corresponding to 10.1% and 41.7% for the full and empty rAAV8 peak areas, respectively. In addition, the full/empty ratio of the rAAV8 viral capsids was also found to increase over time (RSD = 20.5%) when using water as the sample diluent. Conversely, rAAV8 dilutions performed in the formulation buffer (PBS + 0.001% poloxamer 188) resulted in more stable samples over time. More limited variations of the rAAV8 full (RSD = 8.6%) and empty (RSD = 8.3%) peak areas were observed in such dilution conditions, and the measurement of the full/empty ratio was much more precise (RSD = 3.4%). The generic formulation buffer was therefore selected as the preferential diluting solvent, and the remaining rAAV8 analyses were always performed within 3 days from sample preparation to limit variability related to sample stability.

### 2.2. Optimization of the Mobile Phase Conditions

**Figure 1:**
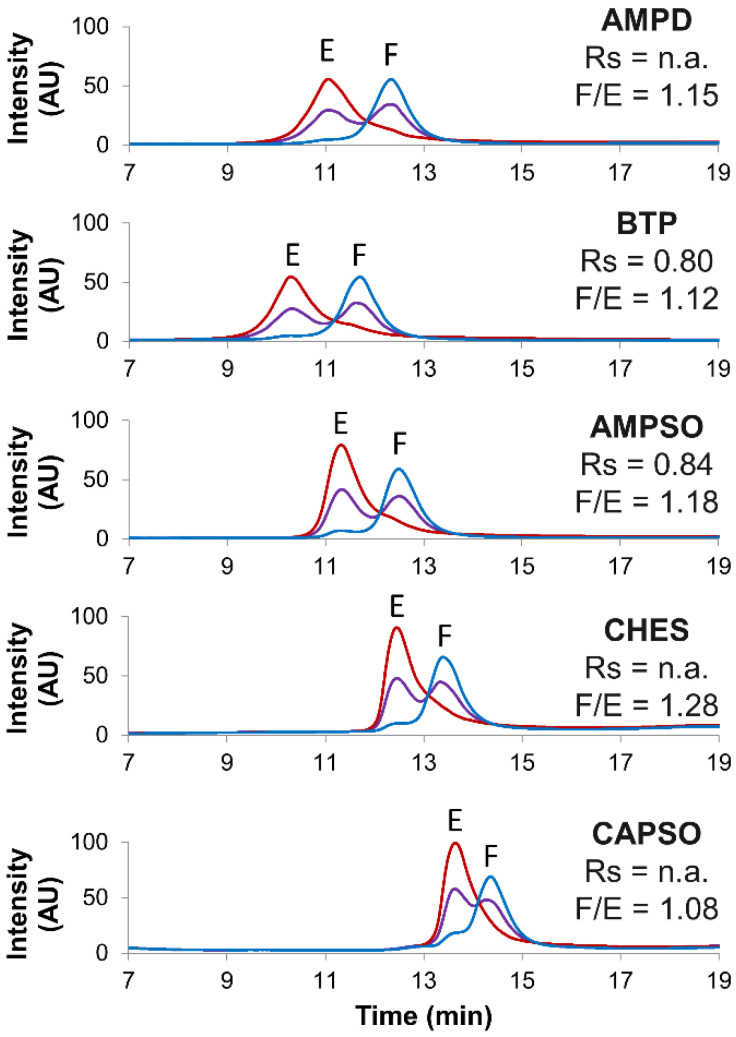
Screening of several buffer types. Empty (red line), full (blue line) and mix of full and empty (violet line) rAAV8 capsids analyzed in AEX mode by using five different buffer types, namely AMPD, BTP, AMPSO, CHES and CAPSO. Gradient conditions: generic linear gradient consisting of buffer pH 9.0 in combination with NaCl as eluent salt. Acronyms legend: E = empty rAAV8 capsid, F = full rAAV8 capsid, Rs = peak resolution, F/E = full/empty ratio, n.a. = not available.

Method development was carried out to further optimize the separation of empty and full rAAV8 capsids. Multiple buffer types, pH and salt types were screened to optimize the gradient conditions. The first optimized parameter was the buffer type. The reference BTP buffer was compared to four alternative biological buffers (Good’s buffers) having different pKa and chemical structures (Table 2), namely AMPD, AMPSO, CHES and CAPSO. The chromatograms obtained with these different buffered mobile phases (pH 9.0) are reported in Figure 1.

The full/empty ratio (F/E) and the chromatographic resolution (Rs) were used as benchmarks to determine the most suitable buffer type. However, no remarkable differences were detected, the full/empty ratios were not significantly affected (ranging from 1.08 to 1.28), and the resolution was only slightly better when using the AMPSO buffer (Rs = 0.84) in comparison to that of the reference BTP buffer (Rs = 0.80).

**Figure 2:**
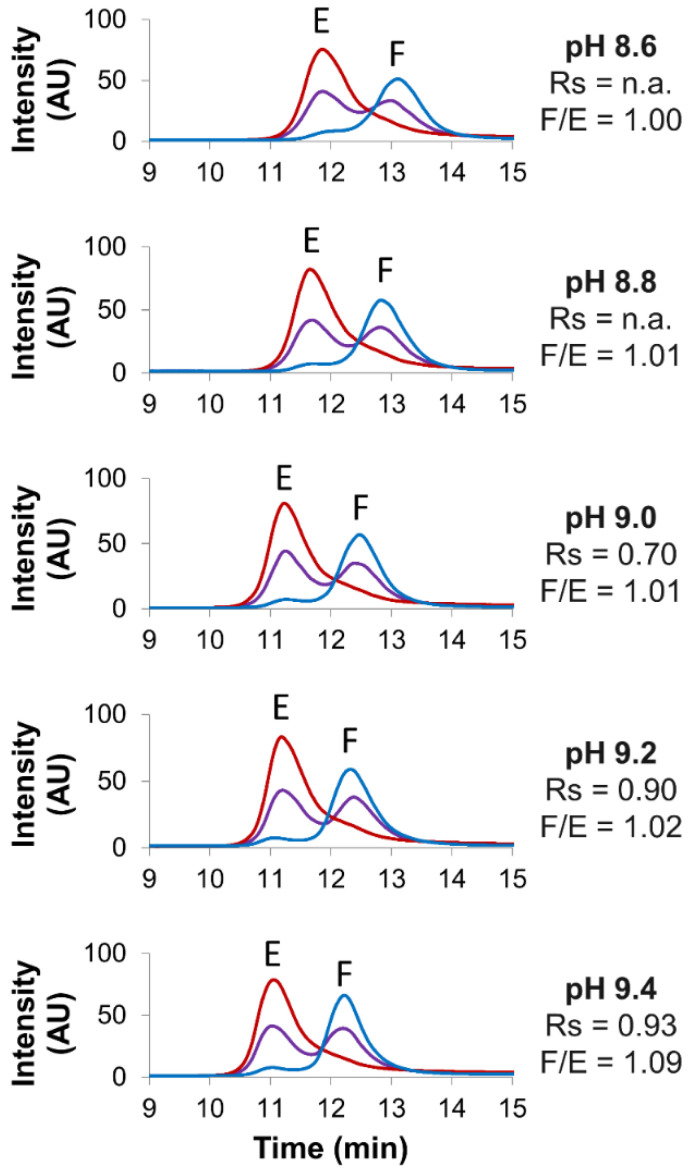
Screening of various pH values. Empty (red line), full (blue line) and mix of full and empty (violet line) rAAV8 capsids analyzed in AEX mode by using five different pH, namely 8.6, 8.8, 9.0, 9.2 and 9.4. Gradient conditions: linear gradient consisting of AMPSO buffer in combination with NaCl as eluent salt. Acronyms legend: E = empty rAAV8 capsid, F = full rAAV8 capsid, Rs = peak resolution, F/E = full/empty ratio, n.a. = not available.

A mobile phase consisting of an AMPSO buffer was then screened at different pH (8.6–9.4, accounting for 0.2 pH unit intervals) to evaluate the impact of this parameter on separation quality. As reported in Figure 2, a slight decrease of retention time was observed when increasing the mobile-phase pH.

This behavior is in line with the AMPSO pKa being equal to 9. When using mobile phases buffered at pH values lower than its pKa (here 8.6 and 8.8), a higher amount of the AMPSO neutral form is present in the solution, while with mobile phases buffered at pH values above the pKa (here 9.2 and 9.4), a higher amount of the negatively charged form of the AMPSO is expected in the solution. As only the negatively charged form of the AMPSO buffer is competing with the analytes for the binding to the positively charged stationary phase, a slight decrease of retention times was observed when increasing the mobile-phase pH. Although the full/empty ratios were not significantly affected by pH variation (ranging from 1.00 to 1.09), a higher resolution was obtained at pH 9.4 (Rs = 0.93) as compared to that at pH 9.0 (Rs = 0.70).

**Figure 3:**
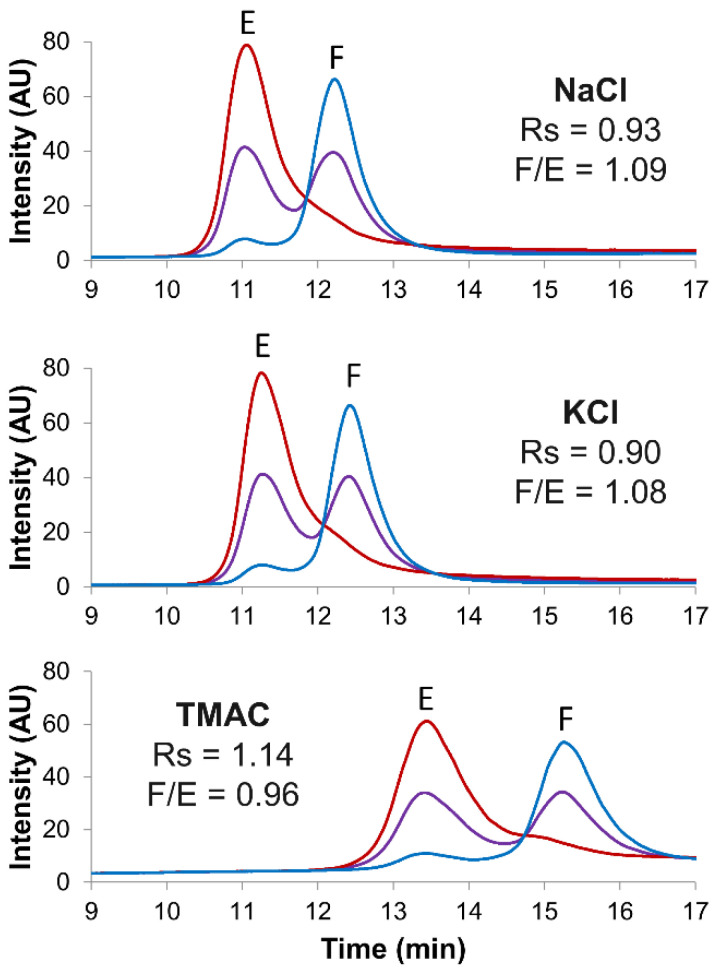
Screening of several salt types. Empty (red line), full (blue line) and mix of full and empty (violet line) rAAV8 capsids analyzed in AEX mode by using three different salt types, namely NaCl, KCl and TMAC. Gradient conditions: linear gradient consisting of AMPSO buffer pH 9.4 in combination with different eluent salts. Acronyms legend: E = empty rAAV8 capsid, F = full rAAV8 capsid, Rs = peak resolution, F/E = full/empty ratio.

Then, three different salt types were screened as eluents for separating full and empty rAAV8 capsids. Figure 3 shows the corresponding chromatograms obtained by using the reference NaCl salt in comparison to those with KCl and TMAC in an AMPSO buffer (pH 9.4). Notably, although the full/empty ratios were not significantly impacted (ranging from 0.96 to 1.09), TMAC allowed a better resolution (Rs = 1.14) in comparison to that with the reference NaCl (Rs = 0.93). Finally, the optimized gradient conditions (AMPSO buffer pH 9.4 in combination with TMAC as eluent salt) were tested at three different mobile phase flow rates, namely 0.7, 0.3 and 0.1 mL/min. As illustrated in Figure S3, 0.3 mL/min was selected as the best compromise between chromatographic resolution, peak intensity and mobile phase consumption.

In the end, it is worth mentioning that despite the exhaustive method optimization, resolution between full and empty rAAV8 capsids was only slightly increasing between “BTP buffer pH 9.0 in combination with NaCl as eluent salt, flow rate 0.7 mL/min”, and “AMPSO buffer pH 9.4 in combination with TMAC as eluent salt, flow rate 0.3 mL/min”. An alternative strategy was therefore applied to boost the separation of the full/empty viral capsids.

### 2.3. Optimization of the Step Gradient Method

#### 2.3.1. Development of a Step Gradient Method

**Figure 4:**
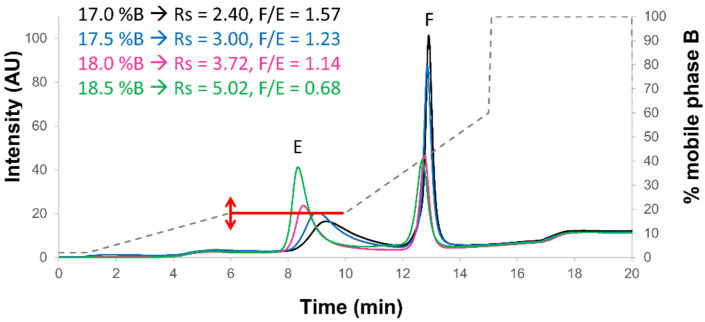
Optimization of the isocratic step volumetric concentration. Mix of full and empty rAAV8 capsids analyzed in AEX mode by using the AMPSO buffer pH 9.4 in combination with TMAC as eluent salt and the step gradient elution mode including a 4n min isocratic step (red line) hold at different % B, namely 17% (black line), 17.5% (blue line), 18% (pink line), and 18.5% (green line). Acronyms legend: E = empty rAAV8 capsid, F = full rAAV8 capsid, Rs = peak resolution, F/E = full/empty ratio.

As described in Section 2.2, it remains difficult to improve selectivity between full and empty capsids simply by tuning the stationary phase and mobile phase under AEX conditions. Therefore, the gradient elution profile was optimized. Indeed, it is well known from the early days of chromatography that large proteins show a specific elution behavior under reversed phase liquid chromatography (RPLC) conditions, where the retention is extremely sensitive to mobile phase composition [27,28]. This particular retention behavior has been described as on/off or bind and elute mechanism, and can be explained as follows: the retention of a large solute is nearly infinite in a weak eluent (this corresponds to the “on” or “bind” state, where the protein species are fully adsorbed at the column inlet), while only a limited increase in eluent strength results in a huge retention decrease (this corresponds to the “off” or “elute” state, as the protein migrates towards the column outlet without any further interactions). In practice, we have recently developed a strategy combining isocratic steps and very short steep gradient segments at protein elution composition, to tune selectivity as desired [29,30]. This strategy was successfully applied to monoclonal antibodies in replacement of linear gradients, even if the latter are often more robust and easier to transfer. To the best of our knowledge, this innovative strategy has only been applied once in AEX for the separation of empty and full capsids of recombinant adeno-associated viruses, and the authors of this work named their strategy “modular discontinuous gradient” [22]. In the present work, this “step gradient approach” was evaluated to improve the separation of full and empty rAAV8 capsids. However, to easily obtain the final step gradient conditions, while avoiding a tedious trial-and-error method development approach, we used the HPLC modeling software DryLab. Based on two initial AEX gradients from 0 to 60% B in 15 and 45 min and the measurement of retention times for the two species, the software was able to establish the retention models of rAAV species and simulate any type of gradient, including potential isocratic steps at a given ionic strength. For the rAAV8 sample, DryLab modeled the following gradient: 2% B held at the beginning of the gradient for 1 min. Then, the gradient increased from 2% to 17.5% B in 5 min, the isocratic step held for 4 min, and then gradient was increased from 17.5% to 60% B in 5 min. Finally, we added a washing step at 100% B for 5 min and an equilibration step of the column at 2% B for 5 min. The optimal conditions suggested by DryLab were experimentally verified, but various isocratic hold compositions around the optimal value were tested (i.e., 17%, 17.5%, 18% and 18.5% B). The corresponding chromatograms were reported in Figure 4. As expected, the complete separation of full and empty rAAV8 peaks was obtained whatever the isocratic step composition. To assess the best conditions, two main figures of merit were evaluated in Figure 4, namely the full/empty ratio and the chromatographic resolution.

When using an isocratic composition of 17% or 17.5% B, the peak corresponding to the empty capsid (first eluted peak) was very broad (insufficient eluent strength and focusing effect). Therefore, the full/empty ratios were too high, probably due to incomplete elution of the empty capsid species (one part of the empty capsid was eluted together with the full capsid). On the other hand, when using 18.5% B, peak shapes were quite good and resolution was maximal (Rs of 5.02), but the full/empty ratio was not in agreement with the theoretical expectations (only 0.68). Finally, when using 18% B, the resolution was still very good (Rs of 3.72), peak tailing/broadening was acceptable, and the full/empty ratio was close to the expected value (1.14). It was particularly interesting to notice that the full/empty ratio could be modified depending on the gradient elution program. This critical aspect has to be taken into account when using the method in a QC environment. Indeed, a small modification of the volumetric composition can already have a significant effect on the final chromatogram (both resolution and full/empty ratios may be affected). This means that the HPLC pumping system has to be highly accurate, which should not be a problem with modern HPLC/UHPLC systems. In the present work, a quaternary pumping system (low-pressure mixing technology) was used, which is known to be less accurate in terms of composition delivery than a binary system (high-pressure mixing system). Therefore, the experiments reported in Figure 4 were not carried out under the best possible conditions offering the highest robustness, but they already proved the applicability of the procedure.

**Figure 5:**
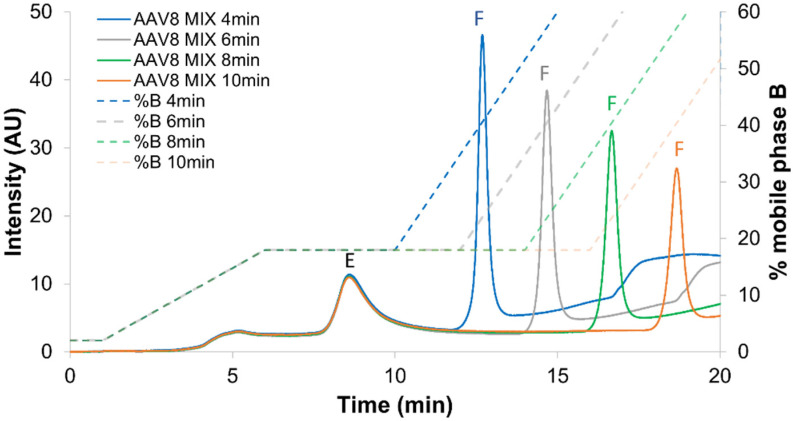
Optimization of the isocratic step duration. Mixture of full and empty rAAV8 capsids analyzed in AEX mode by using the AMPSO buffer pH 9.4 in combination with TMAC as eluent salt and the step gradient elution mode including an isocratic step hold at 18% B for 4 (blue line), 6 (grey line), 8 (green line) and 10 (orange line) minutes. Acronyms legend: E = empty rAAV8 capsid, F = full rAAV8 capsid.

Besides the ionic strength employed at the isocratic step, the duration of the isocratic segment can also be tuned to modify selectivity and resolution. It is indeed important to keep in mind that segment duration will only affect the retention of the next eluting peak (full capsid) and will have no effect on the previously eluted peak (empty capsid). Similarly to what was done for the optimization of volumetric composition, the segment duration suggested by DryLab (4 min) was experimentally tested, but three longer isocratic durations were also evaluated, namely 6, 8 and 10 min. The corresponding data are reported in Figure 5.

**Figure 6:**
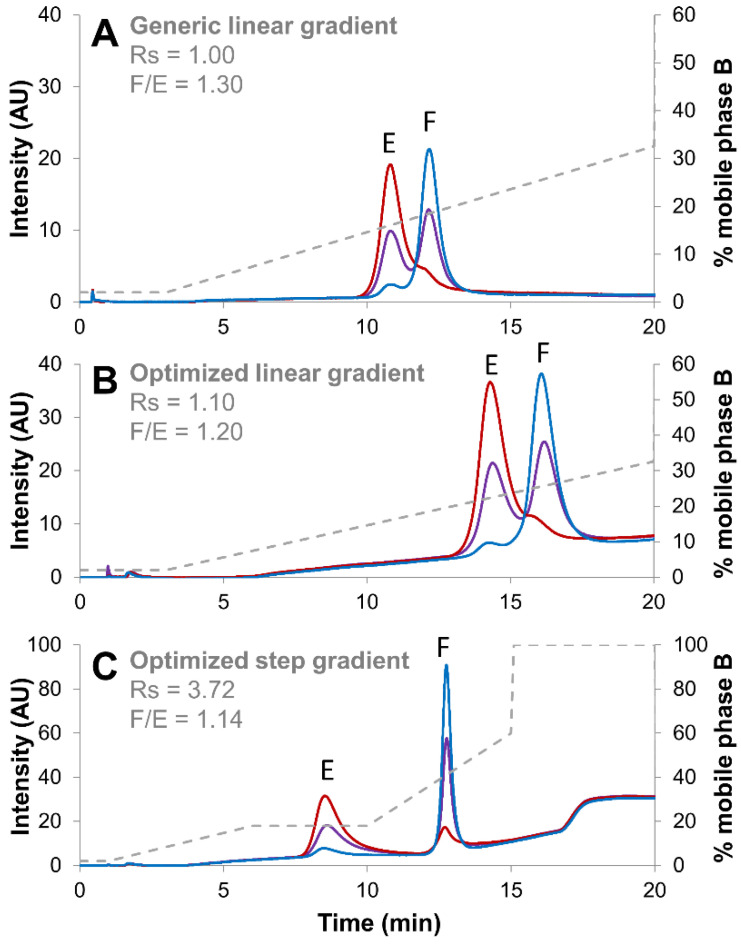
Empty (red line), full (blue line) and mixture of full and empty (violet line) rAAV8 capsids analyzed in AEX mode by using three different elution strategies, namely a generic linear gradient consisting of BTP buffer pH 9.0 in combination with NaCl as eluent salt (**A**), an optimized linear gradient consisting of AMPSO buffer pH 9.4 in combination with TMAC as eluent salt (**B**) and an optimized step gradient including a 4 min isocratic step hold at 18% B (**C**). Acronyms legend: E = empty rAAV8 capsid, F = full rAAV8 capsid, Rs = peak resolution, F/E = full/empty ratio.

When isocratic step duration was too long, a decrease in the peak intensity and peak area corresponding to the full species was observed, while the peak corresponding to empty species was absolutely not affected (Figure 5). This implies that the full species were improperly eluted during the long isocratic step, leading to quantitative loss of these species. This result was reflected by an incorrect full/empty ratio (value too low) after a long isocratic step. Thus, the 4 min isocratic step was selected to avoid this phenomenon. It is also important to notice that a shorter isocratic step was not tested, as we need to maintain a sufficient resolution between the two species. Following the optimization of step gradient conditions, a systematic comparison was conducted between the generic method (Figure 6A), the optimized linear gradient (Figure 6B) and the optimized step gradient method (Figure 6C).

As already discussed in Section 2.2., the gain in terms of chromatographic resolution between chromatograms reported in Figure 6A,B was quite modest, while the ratio of full/empty remained comparable. On the other hand, the resolution improvement was remarkable when applying the step gradient method (Rs of 3.72), while the full/empty ratio remained comparable (1.14). This confirms the interest to develop AEX methods involving the use of a step gradient for rAAV.

#### 2.3.2. Method Validation by Full/Empty Quantification

To check the applicability of the developed step gradient AEX method for the characterization of rAAV8, two different types of samples, including both the full and empty capsids, were obtained from two different providers (i.e., Sirion Biotech and Virovek).

With rAAV8 obtained from Sirion Biotech, the retention time of rAAV8 empty capsids was slightly larger than for the Virovek supplier. This can be explained by a vector production process that leads to a different number of negative charges on the capsid, which may induce somewhat larger retention of the sample. Thus, the separation conditions for full and empty capsids for the rAAV8 from Sirion Biotech were slightly different, and the isocratic step was set at 18.5% for mobile phase B instead of 18.0% to allow adequate elution of the Sirion empty capsids.

**Figure 7:**
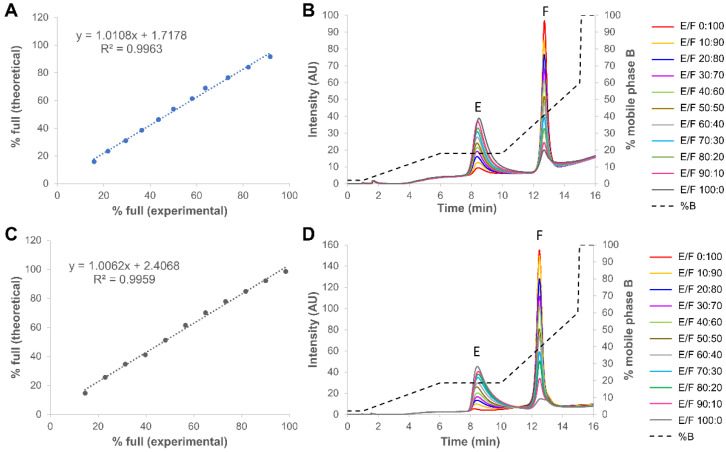
Quantification of percentage of full capsids in different empty and full rAAV8 mixtures obtained from two different providers, namely Virovek (**A**,**B**) and Sirion (**C**,**D**). Gradient conditions: optimized step gradient consisting of AMPSO buffer pH 9.4 in combination with TMAC as eluent salt. Acronyms legend: E = empty rAAV8 capsid, F = full rAAV8 capsid, F/E = full/empty ratio.

For this part of the work, 11 mixtures with both full and empty capsids co-mixed in different percentages (ranging from 0 to 100% of full capsids) in PBS buffer were analyzed. Figure 7 shows the corresponding chromatograms of all co-mixtures, with 0:100 and 100:0 empty/full sample corresponding to the pure full and empty capsids, respectively. As shown in the chromatograms, the full capsids sample from Virovek already contained about 10% of empty capsids, while this value dropped to only 1–2% for the Sirion Biotech rAAV8. On the other hand, the empty capsid samples from the two providers already contained more than 14% of full capsids. In Figure 7, we have also graphically represented the experimental vs. theoretical percentages of full rAAV8 capsids in the mixtures. The experimental values were exclusively obtained from the peak areas. As illustrated, a linear change in the peak area of both peaks (the R^2^ values were higher than 0.995 for the two different samples), with corresponding changes in percent peak areas of empty and full rAAV8 capsids, was monitored. These observations demonstrated that the area of each peak was additive, linear and specific to the empty and full rAAV8 capsids. This confirms that the developed method has the potential to be used in a QC environment, as it is precise, linear and sufficiently robust.

## 3. Materials and Methods

### 3.1. Chemical and Reagents

Bis-Tris propane (BTP, ≥99.0%), 2-amino-2-methyl-1,3-propanediol (AMPD, ≥99.0%), N-(1,1-dimethyl-2-hydroxyethyl)-3-amino-2-hydroxypropanesulfonic acid (AMPSO, ≥ 99.0%), 2-(cyclohexylamino)ethanesulfonic acid (CHES, BioUltra, ≥99.5%), 3-(cyclohexylamino)-2-hydroxy-1-propanesulfonic acid (CAPSO, ≥99% anhydrous basis), magnesium chloride hexahydrate (BioXtra, ≥99.0%), hydrochloric acid solution (1 N), sodium chloride (BioUltra, for molecular biology, ≥99.5%), potassium chloride (BioUltra, for molecular biology, ≥99.5%), tetramethylammonium chloride (TMAC, LiChropur, ≥99.0%) were purchased from Sigma-Aldrich (Buchs, Switzerland). Sodium hydroxide solution (1 N) was obtained from VWR Chemicals. Phosphate-buffered saline (PBS) and poloxamer 188 were obtained from Roche Diagnostics GmbH (Penzberg, Germany). Water was provided by a Milli-Q purification system from Millipore (Bedford, MA, USA).

### 3.2. Sample Preparation

The concentration of rAAV samples was expressed as the number of viral particles per mL (vp/mL). In the present work, rAAV8 samples were obtained either from Virovek (Hayward, CA, USA) at a concentration of 2.00 E + 13 vp/mL or from Sirion Biotech (Graefelfing, Germany) at 5.00 E + 13 vp/mL. In both cases, they were stored at −80 °C. Prior to the analyses, empty and full samples were diluted to 1.00 E + 12 vp/mL in the appropriate solvent (water, phosphate buffer solution with 0.001% poloxamer 188 or mobile phase used for the experiment). For the “mix sample” containing both the full and empty capsids, appropriate volumes of the full diluted sample and the empty diluted sample were taken and mixed to obtain the desired ratio of full and empty capsids in the aliquot. All the samples were analyzed within 72 h and stored at 4 °C after the sample preparation.

### 3.3. Instrumentation and Experimental Conditions

AEX analyses were performed on an ACQUITY UPLC H-Class system (Waters, Milford, MA, USA) equipped with an auto-sampler, including an injection loop of 50 µL, a quaternary solvent delivery pump, and a fluorescence detector (FD). Data were acquired using FLR excitation at 280 nm and emission at 350 nm. Table 1 lists the chromatographic columns investigated in this work that were kept at room temperature during the analyses. Twenty microliters of 1.00 E + 12 vp/mL samples were injected in all cases, corresponding to a column load of 2.00 E + 10 vp. Acquisitions were performed in salt-gradient mode and applied to full, empty, and mixed rAAV8 samples. Data acquisition and instrument control were performed by Empower Pro 3 software (Waters, Milford, MA, USA). Retention and resolution modeling was performed with DryLab^®^ 4 software (Molnár-Institute, Berlin, Germany).

#### 3.3.1. Generic Linear Gradient

Mobile phase A was composed of 65 mM BTP and 2 mM magnesium chloride hexahydrate in water adjusted at pH 9.0 with sodium hydroxide solution 1 M. Mobile phase B was composed of 65 mM BTP, 2 mM magnesium chloride hexahydrate, and 500 mM sodium chloride in water adjusted at pH 9.0 with sodium hydroxide solution 1 M. The generic gradient started with 2% B held for 3 min. Then, the linear gradient was increased from 2 to 56% B in 30 min, followed by a washing step performed at 100% B for 5 min and an equilibration step of the column at 2% B for 6 min. The column was kept at room temperature, and flow rate was 0.7 mL/min unless stated otherwise.

#### 3.3.2. Optimized Linear Gradient

The optimized mobile phase A was composed of 65 mM AMPSO and 2 mM magnesium chloride hexahydrate in water adjusted at pH 9.4 with sodium hydroxide solution 1 M. The optimized mobile phase B was composed of 65 mM AMPSO, 2 mM magnesium chloride hexahydrate and 500 mM tetramethylammonium chloride in water adjusted at pH 9.4 with sodium hydroxide solution 1 M. Flow rate was 0.3 mL/min unless stated otherwise. The same gradient conditions described in Section 3.3.1. were applied.

#### 3.3.3. Optimized Linear Gradient including an Isocratic Step

By using the same mobile phase compositions described in Section 3.3.2., gradient was modified as follows: 2% B was held at the beginning of the gradient for 1 min. Then, the gradient was increased from 2 to 18% B in 5 min, the isocratic step at 18% B was held for 4 min, and then the gradient was increased from 18 to 60% B in 5 min, followed by a washing step performed at 100% B for 5 min and an equilibration step of the column at 2% B for 5 min. The column was kept at room temperature and flow rate was 0.3 mL/min unless stated otherwise.

## 4. Conclusions

This work focuses on the detailed evaluation of AEX as a suitable technique for the determination of the full/empty ratio, a critical quality attribute of rAAV. Before the analysis, it is important to consider the stability of rAAV, which was found to be critical in pure water. Therefore, a mixture of PBS and Poloxamer 188 has to be ideally considered as sample diluent.

In this study, thorough method development was carried out using the rAAV serotype 8 (rAAV8) as a model sample. As highlighted, whatever the type of material (monolith or nonporous silica) and hardware composition (stainless steel or PEEK), the AEX stationary phase had only a negligible impact on the resolution between full and empty capsids, and no partially filled capsids could be detected. Various types of buffers were also investigated (i.e., BTP, AMPD, AMPSO, CHES and CAPSO), and here again, it appeared that the buffers played a minor role in improving chromatographic resolution. Similarly, whenever the mobile-phase pH was around 9 (ranging from 8.6 to 9.4), the separation between full and empty capsids remained almost identical. Finally, three types of salt (TMAC, KCl and NaCl) were tested to elute the rAAV species in AEX, and only a slight improvement was observed with TMAC. In the end, when considering the initial and the fully optimized conditions, resolution between full and empty rAAV8 capsids was only increased from 1.00 (BTP buffer pH 9.0 in combination with NaCl as eluent salt, flow rate 0.7 mL/min) to 1.10 (AMPSO buffer pH 9.4 in combination with TMAC as eluent salt, flow rate 0.3 mL/min), which is clearly too low.

Interestingly, we found that the best way to improve the AEX separation of full/empty capsids consisted of applying a step gradient elution mode to take advantage of the on/off retention behavior of capsids. With these conditions, the gain in terms of chromatographic resolution was radical (Rs of 3.72), while the full/empty ratio remained comparable (around 1.1). This confirms the interest to develop AEX methods involving the use of a step gradient for rAAV.

Finally, thanks to the baseline separation of full/empty capsids, we have evaluated the method from a quantitative perspective. We found that the area of each peak was additive, linear and specific to the empty and full rAAV8 capsids. This confirms that the developed method is suitable for a QC environment. In the future, the analytical strategy may also be applied to other serotypes.

## 5. Patents

A patent application corresponding to the content of this manuscript has been filed and is pending.

## Figures and Tables

**Table 1:** List of investigated chromatographic columns and their properties (chemistry: quaternary ammonium for all columns). PEEK and SS stand for polyether ether ketone and stainless steel, respectively.

Acronym	Name	Provider	Column Hardware	Column Dimensions (mm)	Particle Size (µm)
PP	ProPac SAX-10	Thermo	Nonporous PEEK	50 × 4	10
PS	ProSwift SAX-1S	Thermo	Monolith PEEK-lined SS	50 × 4.6	-
AX	Bio-SAX	Agilent	Nonporous PEEK	50 × 4.6	5
QS	TSKgel Q-STAT	Tosoh	Nonporous SS tubing, PEEK frits	100 × 4.6	7

**Table 2:** List of investigated buffers and their chemical structures.

Buffer Name	Acronym	MW (g/mol)	pK_a_ (25 °C)	Structure
2-Amino-2-methyl-1,3-propanediol	AMPD	105.15	8.8	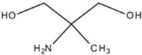
1,3-Bis[tris(hydroxymethyl)methylamino]propane	BTP	282.33	9.0	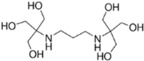
N-(1,1-Dimethyl-2-hydroxyethyl)-3-amino-2-hydroxypropanesulfonic acid	AMPSO	227.28	9.0	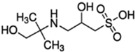
2-(Cyclohexylamino)ethanesulfonic acid	CHES	207.29	9.5	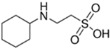
3-(Cyclohexylamino)-2-hydroxy-1-propanesulfonic acid	CAPSO	237.32	9.6	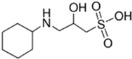

## Data Availability

Not applicable.

## Supplementary Materials

The following supporting information can be downloaded at: https://www.mdpi.com/article/10.3390/ijms232012332/s1.
