# Supplementary material for: Anion-Exchange Chromatography at the Service of Gene Therapy: Baseline Separation of Full/Empty Adeno-Associated Virus Capsids by Screening of Conditions and Step Gradient Elution Mode
Source: Int J Mol Sci. 2022 Oct 15;23(20):12332. doi: 10.3390/ijms232012332 (PMC9604245; doi:10.3390/ijms232012332)

Supporting Information

TABLE OF CONTENTS

Figure S1 Comparison of different column hardware…………………………………… S1

Figure S2 Sample stability study over 7 days…………………………………………. S2

Figure S3 Screening of AEX analysis flow rate……………………………………….. S3

Figure S1. Comparison of different column hardware. Empty (red line), full (blue line) and mixture of full and empty (violet line) rAAV8 capsids analysed in AEX mode by using 4 different column hardware, namely PP (A), PS (B), AX (C), and QS (D). Gradient conditions: reference linear gradient consisting of BTP buffer pH 9.0 in combination with NaCl as eluent salt. Acronyms legend: E = empty rAAV8 capsid, F = full rAAV8 capsid, Rs = peak resolution, F/E = full/empty ratio.

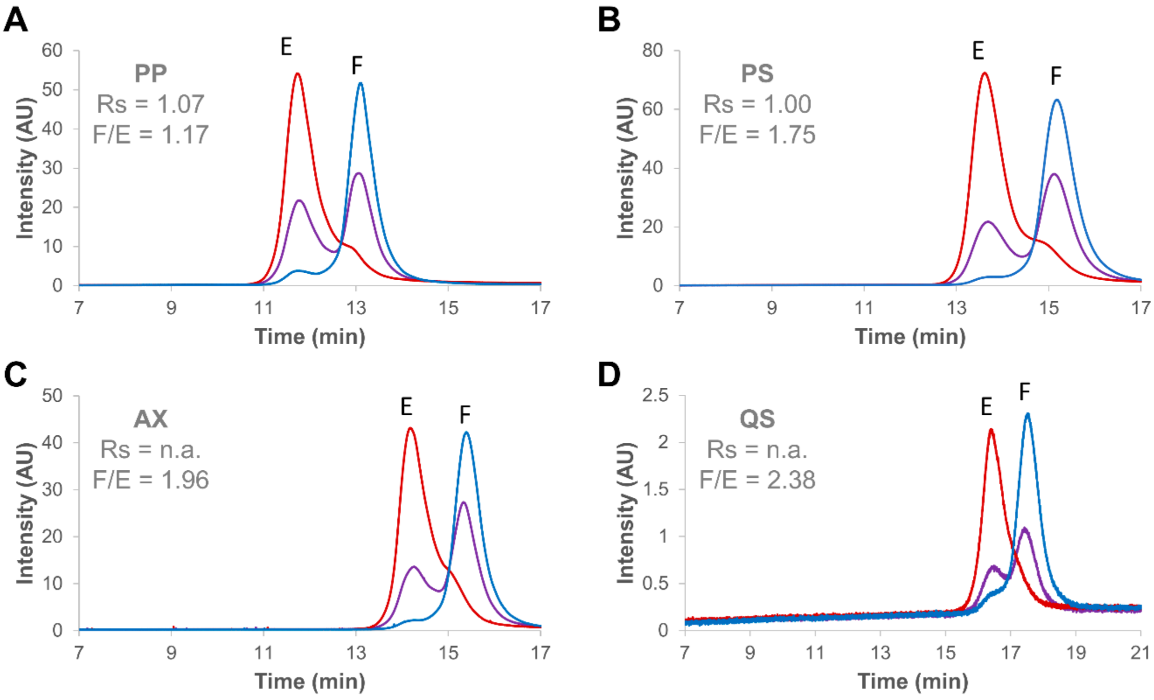

Figure S2. Sample stability study over 7 days. Samples consisting of full/empty mixture (panels A and B), full (panels C and D), and empty (panels E and F) rAAV8 capsids were diluted in water (panels A, C, E) and PBS buffer enriched with 0.001% poloxamer 188 (panels B, D, F) and analyzed at 0 (blue), 1 (green), 3 (orange), and 7 (yellow) days after dilution. Acronyms legend: E = empty rAAV8 capsid, F = full rAAV8 capsid.

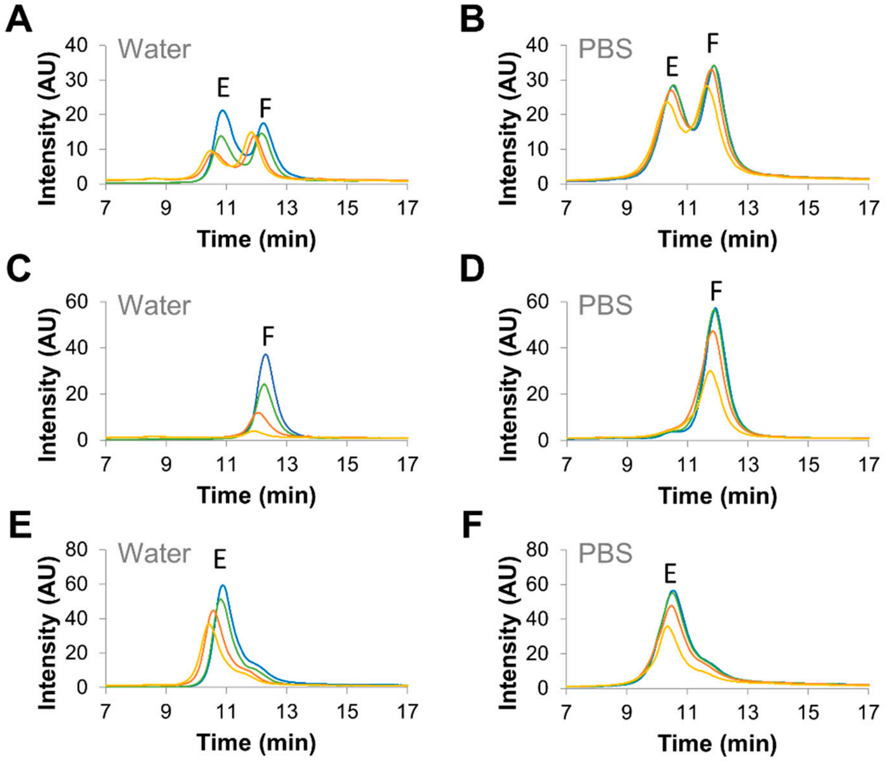

Summary of the chromatographic parameters related to the rAAV8 stability study:

	t_R empty (min)	t_R full (min)	F/E ratio	Area empty (AU)	Area full (AU)	Resolution
**Dilution in water**						
**Day 0**	10.88	12.23	0.89	21.21	17.55	1.03
**Day 1**	10.83	12.17	1.06	13.85	14.62	1.09
**Day 3**	10.60	11.95	1.37	9.11	14.04	1.08
**Day 7**	10.47	11.84	1.38	9.60	15.02	1.08
**SD**	0.19	0.18	0.24	5.60	1.55	0.03
**Average**	10.69	12.05	1.18	13.44	15.31	1.07
**RSD (%)**	1.8%	1.5%	20.5%	41.7%	10.1%	2.5%
**Dilution in PBS buffer**						
**Day 0**	10.51	11.87	1.09	28.40	34.24	n.a.
**Day 1**	10.55	11.87	1.09	28.56	34.18	n.a.
**Day 3**	10.47	11.78	1.12	27.01	33.03	n.a.
**Day 7**	10.32	11.64	1.17	23.75	28.38	n.a.
**SD**	0.10	0.11	0.04	2.23	2.78	/
**Average**	10.46	11.80	1.12	26.93	32.46	/
**RSD (%)**	0.9%	1.0%	3.4%	8.3%	8.6%	/

Figure S3. Screening of mobile phase flow rate. Mixture of full and empty rAAV8 capsids analysed in AEX mode by using three different flow rates, namely 0.7 mL/min (grey line), 0.3 mL/min (green line), and 0.1 mL/min (orange line). Gradient conditions: optimized linear gradient consisting of AMPSO buffer pH 9.4 in combination with TMAC as eluent salt.

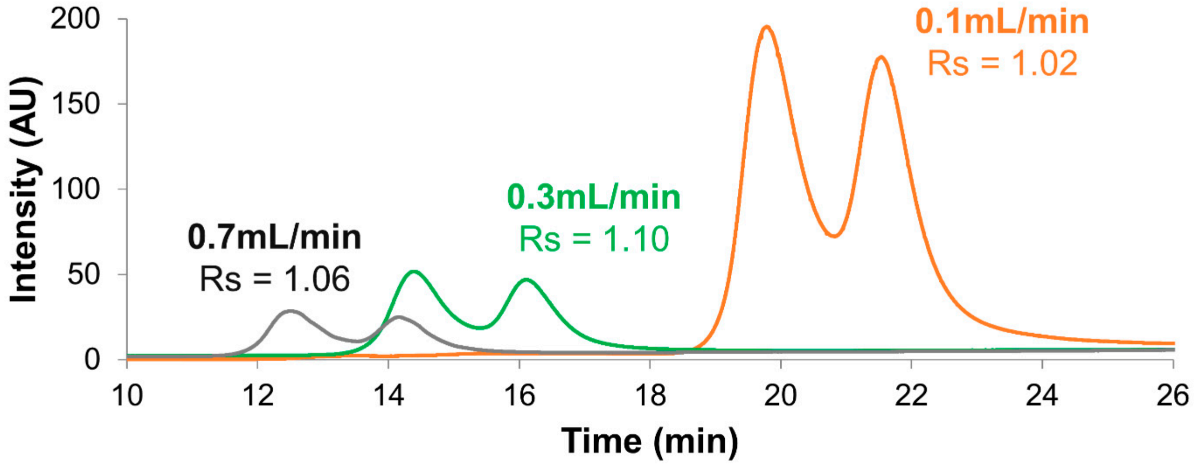

Supplement: Supplementary file 1 [file ijms-23-12332-s001.zip › ijms-1945264-supplementary.pdf]
